# Social media use and social connectedness among adolescents in the United Kingdom: a qualitative exploration of displacement and stimulation

**DOI:** 10.1186/s12889-021-11802-9

**Published:** 2021-09-24

**Authors:** Lizzy Winstone, Becky Mars, Claire M. A. Haworth, Judi Kidger

**Affiliations:** 1 Population Health Sciences, Bristol Medical School, University of Bristol, Bristol, BS8 2BN UK; 2 NIHR Biomedical Research Centre at the University Hospitals Bristol NHS Foundation Trust and the University of Bristol, Bristol, BS8 2BN UK; 3 School of Psychological Science, University of Bristol, Bristol, BS8 1TU UK; 4 The Alan Turing Institute, British Library, London, NW1 2DB UK

**Keywords:** Social media, Social connectedness, Adolescence, Peer relationships, Family relationships

## Abstract

**Background:**

Connectedness to family and peers is a key determinant of adolescent mental health. Existing research examining associations between social media use and social connectedness has been largely quantitative and has focused primarily on loneliness, or on specific aspects of peer relationships. In this qualitative study we use the displacement hypothesis and the stimulation hypothesis as competing theoretical lenses through which we examine the complex relationship between social media use and feelings of connectedness to family and peers.

**Methods:**

In-depth paired and individual interviews were conducted with twenty-four 13–14-year-olds in two inner-city English secondary schools. Interviews were transcribed verbatim, coded and thematically analysed.

**Results:**

Analysis identified four themes: (i) ‘Displacement of face-to-face socialising’ (ii) ‘Social obligations’ (iii) ‘(Mis)Trust’ and (iv) ‘Personal and group identity’. Results indicated stronger support for the stimulation hypothesis than the displacement hypothesis. We found evidence of a complex set of reciprocal and circular relationships between social media use and connectedness consistent with a ‘rich-get-richer’ and a ‘poor-get-poorer’ effect for family and peer connectedness – and a ‘poor-get-richer’ effect in peer connectedness for those who find face-to-face interactions difficult.

**Conclusion:**

Our findings suggest that parents should take a measured approach to social media use, providing clear guidance, promoting trust and responsible time management, and acknowledging the role of social media in making connections. Understanding and sharing in online experiences is likely to promote social connectedness. Supporting young people to negotiate breathing space in online interactions and prioritising trust over availability in peer relationships may optimise the role of social media in promoting peer connectedness.

**Supplementary Information:**

The online version contains supplementary material available at 10.1186/s12889-021-11802-9.

## Introduction

Social connectedness is defined as feelings of belonging and closeness to others, as well as satisfaction with relationships and perceived support and opportunities for self-disclosure of personal information. It comprises different domains (peer, school, family and community/ neighbourhood) and is a key social determinant of adolescent mental health and well-being [1–3]. Family connectedness in particular has been found to buffer the negative effects of bullying and to be related to lower risk for suicide-related outcomes and depressive symptoms [3, 4].

Social media use (SMU) is thought to have both positive and negative influences on the lives of young people, for whom it has become an integral part of daily life [5]. In 2018, 80% of 14-year-olds in the United Kingdom (UK) had a profile on a social media or messaging app [6]. For the purposes of this study, we include within social media social network sites as defined by boyd and Ellison [7], in addition to web-based messaging and microblogging services (such as WhatsApp and Tumblr) and social video platforms (such as YouTube). SMU has various functions, with users typically seeking entertainment, communication, inspiration and information. The use of social media to engage with others, either through direct communication or through the publication or consumption of content and its associated feedback, makes it an inherently social part of adolescence [8]. As such, SMU may have important implications for increasing connectedness with individuals and groups [9]. However, concerns have been raised by parents about screen-time interfering with other activities that may also be beneficial to connectedness [10], such as schoolwork, extra-curricular activities and engaging with others face-to-face. Through these competing processes of stimulation and displacement, SMU may simultaneously enhance and undermine social connectedness in adolescence [9].

### The displacement and stimulation hypotheses

The displacement hypothesis was formulated on the basis of internet use rather than social media specifically [11]. The theory of displacement is two-fold, regarding both time displacement and displacement of strong social ties with weak ones. Use of the internet for entertainment purposes – as a solitary, socially disengaged activity comparable to passive consumption of social media content without active engagement – is thought to displace time spent socialising with others offline, subsequently undermining social connectedness [11, 12]. Where used for communication purposes, online engagement and expansion of social networks were thought to be primarily with weak ties rather than with close family and friends, and as such, of little benefit to psychosocial well-being [11, 13].

In line with this hypothesis, previous research has found that SMU is associated with increases in bridging but not bonding social capital, whereby vast expansion of social networks made possible through SMU enhances the number of weak social ties rather than improving relationships with close friends and family [5]. SMU may also displace time spent on other activities beneficial to well-being, including physical exercise and sleep [14, 15]. With regards to family connectedness, an intensive longitudinal experience sampling study found little evidence that time spent using digital technology displaced time spent engaging offline with parents or resulted in problematic parent-adolescent offline interactions [16].

Evidence also exists in support of an opposing theory, the stimulation hypothesis, whereby SMU enhances the user’s existing social resources through increased contact and maintenance of relationships [17, 18]. In direct contradiction to the displacement hypothesis (whereby strong social ties are displaced with weak ones) it has been suggested that adolescents are increasingly using social media to enhance the quality of existing friendships rather than seeking out new connections, leading to beneficial impacts on social connectedness and social and emotional support [17].

One study directly compared the two competing hypotheses and found that, rather than displacing time spent offline with friends, use of instant messenger was positively related to face-to-face socialising, in turn predicting better friendship quality and well-being [19]. This effect was specific to using instant messenger to communicate with friends and did not apply to use of chat rooms (primarily with strangers). The authors suggested that features of online communication – including asynchronous responding and absence of nonverbal cues or responses –could lower social inhibition and encourage sharing of personal information. These intimate self-disclosures can be beneficial to well-being and peer connectedness through enhancing feelings of support and trust [20, 21].

Using experience sampling methodology, researchers have explored fluctuations in adolescents’ use of Instagram, Snapchat and WhatsApp with and without close friends [8]. Findings illustrated the complexity of the relationship between SMU and friendship closeness, with substantial differences at the within- and between-person level. Those who used Instagram or WhatsApp in the previous hour (whether with or without close friends) reported feeling slightly less close to close friends, however, those with a higher average frequency across a three-week period felt closer to their friends than those with less frequent use. Snapchat use was not found to be related to friendship closeness at either the within or between person level [8]. These findings were echoed in a study showing evidence for the displacement hypothesis at the within-person level – with increases in smartphone communication on a particular day reducing face-to-face interaction for a given individual – but not the between-person level – with no discernible difference in the level of face-to-face interaction for more or less prolific online communicators [22].

There is also evidence to suggest the relationship with SMU may be curvilinear, with only excessive levels of SMU found to be associated with lower levels of social capital [20, 23] or poorer psychosocial functioning [24]. In a longitudinal study of adolescents in Belgium [23], Wang et al. found that low to moderate levels of active public Facebook use (that is broadcasting content publicly but not direct messaging with others) were associated with decreased loneliness over time, supporting the stimulation hypothesis. However, higher levels of broadcasting were associated with increased loneliness over time, indicating support for the displacement hypothesis. This indicates that rather than being mutually exclusive theories, both the stimulation and displacement hypotheses may be possible, depending on both the amount and type of SMU [17].

### Objectives of the current study

Most existing research examining the displacement and stimulation hypotheses has been quantitative and has focused primarily on peer connectedness. Qualitative research, and research exploring the relationship between adolescent SMU and family connectedness is scarce. This qualitative study aimed to examine the relationship between SMU and social connectedness (encompassing peers and family) through the experiences and perspectives of a sample of 13–14-year-olds in south-west England.

## Methods

### Participants

Thirteen interviews were conducted with 24 Year 9 students aged 13–14 years (19 girls and five boys) in February–March 2020. Interviews took place at two English secondary schools in inner-city locations. One was in a particularly deprived area with an ethnically diverse and lower socio-economic status student population (measured by the proportion of students eligible for free school meals). The other was a single sex girls’ school with a higher-than-average socio-economic status population. Heads of Year 9 in each school were asked to advertise the study to all classes in the year group, with participant information sheets provided. The information sheets encouraged students with a range of social media experiences to take part, including those who considered themselves to be non-users. Two participants presented themselves as non-users of social media, describing their use of YouTube solely for entertainment purposes. Students volunteered to take part and all volunteers were selected for interview providing they returned signed parental consent forms by a cut-off date. In advance of the interviews, participants indicated on consent forms their preference for participation in an individual or paired interview with a friend (Table 1). Participants received a £10 Amazon voucher by way of thanks.

**Table 1:** Interview format overview

Interview format	Participants
Individual	F3; F10; M4
Paired	F1 & F2; F4 & F5; F6& F7; F8 & M1; M2 & M3; M5 & F9; F11 & F12; F16 & F17; F18 & F19
Group of three	F13, F14 & F15

### Design and procedure

Interviews were all conducted face-to-face by LW. LW is a female PhD student with some previous experience of conducting in-depth interviews with adults, and is trained in qualitative data analysis and conducting research with young people. JK oversaw the process and is a female academic with extensive qualitative research experience. The interviews were audio recorded, took place at school during lesson time and lasted between 45 min and an hour. A topic guide (available in Additional file 1) was used to ensure consistency in covering a number of core areas for discussion, including typical apps and activities used, family and school rules regarding SMU, online interactions with peers, family or strangers and SMU in those experiencing poor mental health. The guide was developed following consultation with a young people’s advisory board – a group of 11–18-year-olds with experience in advising on the design of health-related research materials. The group provided input into issues they felt most important relating to SMU and mental health. Flexibility in the topic guide allowed interview participants to take the conversation in any direction they felt to be relevant to the broad issue of social media and mental health, reflecting on their own SMU as well as that of their peers. This flexibility was felt to be important in mitigating the impact of the adult researchers’ preconceptions about adolescent SMU, social connectedness and mental health, enabling openness to experiences recognised as meaningful by participants themselves.

### Data analysis

An inductive, reflexive approach to thematic analysis was used from a critical realist (contextualist) perspective [25]. SMU is so intertwined with one’s experience and perceptions of interpersonal and intergroup relationships that it would not make sense to refer to there being an authentic truth or reality. However, as researchers aspiring to improve public mental health through recommendations to stakeholders, we need to acknowledge young people’s experiences and feelings as an external reality, whilst recognising the prisms through which these are encountered by young people and interpreted by ourselves [25]. We interpreted the data as adults who experienced adolescence in a time before social media existed and we reflected on this throughout the analytic process.

Notes were taken during and after each interview. These were not coded but used in reflection during analysis. The interviews were transcribed verbatim and imported into NVivo version 12 for coding by LW. Analysis was conducted primarily by LW, from the perspective of an adult using social media for direct communication with existing friends and family. LW acknowledges that her own experiences – both positive and negative – of SMU will unavoidably frame her interpretation of the data.

A systematic and inclusive coding process was adopted, with coding applied flexibly to include unexpected data [25]. Codes were both descriptive (e.g., ‘nothing to do’) and interpretative (e.g., ‘privacy concerns’). Following coding of the complete dataset, initial themes were constructed and reviewed iteratively when examined against each interview, with thematic boundaries altered as necessary. Further amendments were made where appropriate following group discussion between the authors to review both codes and themes. During the review process codes considered irrelevant to the research question (e.g., ‘apps’) were discarded or included within other codes where appropriate (e.g., ‘memories’ was encapsulated within ‘friendships’). Two previously separate themes – ‘keeping in touch’ and ‘time displacement’ were merged into ‘displacement of face-to-face socialising’. Once themes were developed, they were examined in relation to the displacement and stimulation hypotheses to see whether findings confirmed, contradicted, or developed these theories. Participants were not asked to provide feedback on the findings.

## Results

Four themes were identified through analysis of the qualitative interview data with regard to SMU and social connectedness. Table 2 provides an overview of these themes and sub-themes with key illustrative data extracts. Each sub-theme is discussed in turn, along with implications for displacement and stimulation theories. There were no systematic differences in opinions and experiences between participants from the two different schools or those interviewed individually compared to with others, so comparisons are not presented here.

**Table 2:** Overview of themes, sub-themes and illustrative quotes

Theme	Sub-theme	Illustrative Codes	Illustrative quotes
Displacement of face-to-face socialising	Socialising with family	Family dynamics; Keeping in touch	*I have family [abroad] and, like, I have my mum’s cousin’s daughters on Instagram and I just like it and comment and stuff. But my mum connects with her mum…but it’s like money… …We have this group chat with loads of family, like, around the whole world and we always talk, if there’s something happening, events and stuff. (F3)*
	Socialising with friends	Nothing to do; Meet new people	*I guess it’s just like them being there, it’s not like we have to talk every day, but you can once in a while talk with them and it’d still be the same as primary…. And if it wasn’t for social media, we probably wouldn’t be as close as we were and are. (F12)*
Social obligations	Obligation to be available	Being available; Multitasking	*Sometimes it does feel a bit like a job, it’s like, “Right, got to check it now, got to do this.” Sometimes I don’t even want to but I just have to. Again, I don’t want an argument to happen if I’ve ignored someone for like a week because I just didn’t want to open their message or anything like that. (F15)*
	Obligation to provide positive feedback	Responding to pictures; Friendships	*There was one situation that I did not see his [a friend’s] picture, because it did not load up for me. Then the next day he texted me, ‘Oh, can you like for me?’ I was like, ‘What picture?’…Then he started to get angry at me, because he thought I was just airing [avoiding] him, but I actually did not see it, because the picture did not load up for me. (M4)*
(Mis)Trust	Opportunities for adults to demonstrate trust	Parents trust not to do anything bad; Not telling the truth	*When I first got a phone, my mum would say stuff like, “Don’t talk to people you don’t know,” or “Stay away from bad things,” and all that. Since then, I don’t think she has ever said anything about it. She’s kind of set ground rules. Then, now she kind of trusts me to not use it for anything bad or anything like that. (M1)*
	Self-disclosure and fear of screenshotting	Privacy concerns; Online-offline communication	*I don’t like texting when it’s serious, because I know people can screenshot that… I’m just like, “I don’t trust you.” (F2)*
Personal and group identity	Sense of belonging	Interests; shared experiences	*If you’re on five different group chats or if you’re texting people, you know you’re a part of a bigger community, because people want to get involved with you and they want to talk to you, so you feel more social... (M4)*
	Generational disconnect	Adults can’t relate; Group differences	*…they feel like I’m not spending enough time with them and I feel like parents, because they can’t relate to it, with most kids and stuff they’re meant to relate to what happened to them when they’re growing up. Like, “Oh yes, that happened to me, don’t worry.” With phones obviously they didn’t have them when they were growing up so they can’t relate to it and they don’t know what it’s going to be like in the future or how we’re going to be impacted from it… (F15)*

### Displacement of face-to-face socialising

Elements of both displacement and stimulation were interwoven in discussions of online and offline socialising. Participants’ own SMU was sometimes felt to displace face-to-face social activities that promote feelings of connectedness. However, online peer interactions frequently took place when in-person socialising was not possible, helping to alleviate feelings of boredom and loneliness. Social network expansion was also highlighted as a key benefit of SMU, meeting new people, and maintaining contact with old friends outside of school and family members abroad.

#### Socialising with family

Several participants suggested that time spent socialising with family members would likely increase if they were to reduce their social media screen-time. Participant F5 spoke about recently breaking her phone and noted the positive impact it had on increasing time spent with her family.

Those participants who appeared to be minimal social media users reflected most on the importance of not ‘missing out’ on time with family (M3). Being with and ‘helping’ (M2) family was highlighted as a priority for these participants, with M2 suggesting that ‘talking to your family… is safer than talking on social media’. The strong family connectedness depicted by these participants appeared to have a protective effect against SMU displacing time spent together.

More frequently however, references to family connectedness and screen-time suggested increased SMU was a result rather than a cause of poor connectedness. Several participants alluded to their SMU at home as a means of reducing boredom or loneliness, because family members were not available to share meals or converse (F1, F2, M4, F6, F7, F12, F16). Some participants described family situations where disruption to home life, unsocial family dynamics or parents’ work patterns created time where they were left alone and turned to SMU because there was ‘nothing else to do’ (F1, F18, F19). F7 described sitting alone to eat dinner while using her phone and noted ‘…we just don’t do things as a family. It’s not social media, it’s just like, we just don’t do things.’

Consistent with the stimulation hypothesis, participants explained the benefits of communicating via social media to stay connected to family members who did not live close by, enhancing family connectedness beyond the nuclear family unit. This was felt to be particularly beneficial to those who would otherwise find the cost of overseas communication prohibitive. For some, this applied to one-to-one relationships with cousins of a similar age (F8). For others, group chats on social media enabled geographically disparate family members to come together as one to catch up or celebrate special occasions (F3).

Rather than displacing time spent socialising face-to-face with family, these comments showed how SMU was an important means of maintaining social interaction when in-person contact was not possible.

#### Socialising with friends

Across the sample, there were diverging opinions as to whether there was a difference between online and face-to-face socialising. In line with the displacement hypothesis, some found socialising through social media to be less rewarding than face-to-face, pointing particularly to the more genuine feel to in-person interactions where ‘you’ll see how they really are in person’ (F4) and can ‘gauge more’ (F6). Others gave opinions more aligned with the stimulation hypothesis, whereby online socialising facilitated offline interaction when they felt their own personalities to be ‘shy’ or socially ‘awkward’ (F6) – a social compensation effect [26]. As participant F10 put it, ‘I’m better friends with people because I’ve spoken to them more online and therefore in real life, we’re better friends. I wouldn’t say it’s different, no’.

As with family interactions, some participants pointed to the possibility of excessive SMU displacing time spent socialising in person with friends, leading some young people to ‘distance themselves from family and friends’ (M5). Some participants expressed a desire to reduce their own SMU to spend more time ‘meeting up with those people’ they were communicating with online (F5).

However, those who were more frequent users again indicated that online interactions generally replaced face-to-face out of necessity. Friends who were unable to socialise in person due to geographical constraints turned to social media to maintain peer-to-peer interaction. This applied to ‘long-distance friendships’ (F16), keeping in touch with friends at other schools, and those who did not live within walking distance to their close friends (F18).

*Yes, that’s one of the reasons I use social media so much, it’s because all of my friends live so far away. I think my closest friend lives a 20-minute drive from me. So, I use social media to stay in contact with everyone, because that’s the only way you can really talk to people. (F19)*

SMU was thus felt to strengthen or maintain peer connectedness for those with reduced opportunities for offline socialising, providing a protective effect from the risks of poor peer connectedness or loneliness.

SMU also promoted continuity of social networks, enabling young people to stay ‘connected’ (F12) with old friends from primary school, those who have moved away from the area, and friendships formed from extracurricular activities (F10). Without social media, there was a perceived risk that such ‘friendship[s] would just die’ (F16). Rather than ‘weak ties’ of vast online networks suggested by the displacement hypothesis [11], these were presented as close friendships whose enduring existence was stimulated by SMU in the absence of opportunities for offline interaction.

What social media is used for may influence whether it is perceived by the user to be displacing time spent with peers. Participant M1 pointed to the difference between playing PlayStation with a headset on, ‘talking to your friends… you are playing but also talking, so I feel more social, more talkative’ and ‘when you are using social media [passively], you feel isolated. You are just on your phone’ (M1). Whereas passive or excessive SMU may be perceived to displace face-to-face interaction, using social media explicitly for socialising may fulfil more of a stimulation function – enabling friends to ‘hang out’ (M4, M5) online when doing so in person is not possible.

In addition to maintaining stability within their social circle, SMU was also often credited with expansion of participants’ social circle through making new friends online, both by offering opportunities for new introductions, such as through a ‘mutual friend’ (F5), and by facilitating the development of friendships through initial online communication, which felt less intimidating than new face-to-face socialising (M4). Supporting the online enhanced self-disclosure or social compensation hypothesis [26] this was found to be particularly helpful to F6, who described herself as ‘shy’.

*I feel like it’s easier for me because I feel like… if I’d just met someone, like, if someone just came to school now and we had to be friends, I feel like it would take me a while to, like, be able to talk to them without feeling… It depends. I think sometimes I’m really shy, and I think sometimes I’d rather just get to know someone online first… (F6)*

As such, SMU was determined to be an important – and in some cases, critical – means of maintaining and expanding the size of young people’s social networks. SMU was explicitly credited by some participants for network expansion over and above friendship closeness. The network enhancing benefits they ascribed to SMU may be aligned to some extent with the ‘weak ties’ suggested by the displacement hypothesis, bringing limited psychosocial reward [11, 13]. However, many young people in our sample referred to most of their online interactions and relationship maintenance being with existing close friends, illustrative of a stimulation effect. The importance of network size for well-being may vary across individuals, and a discussion between participants F6 and F7 noted the distinction between small numbers of ‘deep’ friendships (F6) that traverse online and offline worlds, and the tendency for some young people (including themselves at an earlier age) to place importance on having ‘loads of followers’ (F6) who were ‘fake friends’ (F7).

### Social obligations

Whilst SMU enhanced feelings of connectedness through enabling participants to keep in touch with others, this was frequently accompanied by demanding expectations amongst peers. Participants reported feeling obliged – in line with social norms – to respond promptly to messages from peers and to provide positive feedback on peers’ social media posts. This was sometimes accompanied by feeling overwhelmed by multiple messages or group chats, guilt associated with making excuses for unavailability or outright peer conflict if expectations were not met. Rather than displacement or stimulation, we suggest this represents a situation of ‘over-stimulation’.

#### Obligation to be available

Most participants reflecting on their own active use of social media made implicit reference to a social media etiquette developed by their generation, to which they either adhered or chose to ignore. Participants felt they were expected to respond immediately to social media messages. Being available to take part in multiple online conversations simultaneously was felt to be ‘stressful’ and ‘a mess in my mind’ (M4), with ending conversations causing difficulty for several participants.

*For me, it is actually hard, because I don’t have a way to end the conversation. I just go on another application and then after an hour or two, I check what they actually wrote and then they’re like, ‘Oh, you came back,’ and then I have to tell them a random explanation. I was like, ‘Oh, I went do something,’ or something, because I don’t want to tell them, ‘Oh, I couldn't be bothered to talk to you anymore,’ because that’s, kind of, a harsh way. (M4)*

Participants often discussed their online communication in obligation-related terms as a means of avoiding conflict with peers, or as a chore necessary to adhere to social norms.

*People don’t assume, “Oh, they're busy.” If my friend didn’t reply to me for a day, I’d instantly think, “Oh, have I done something wrong?” because I feel like a day is quite a long time to go without social media for us, so I’d just be like, “Oh, are they annoyed at me?” (F12)*

This narrative of obligation or duty was underlined by terminology used by participants who reflected on the need to provide peers with an ‘excuse’ (F12, F15) to end online conversations or not to respond immediately to messages, with one participant ‘panicking’ (F14) when her phone was broken in case friends took offense to her lack of contact.

This aligns with neither the displacement nor stimulation hypothesis. These participants seemed to reveal a sense of ‘over-stimulation’ or ‘hyper-connectedness’ with peers, whereby perceived excessive or duty-bound online communication no longer enhanced friendship quality but became a burden attached to friendships.

#### Obligation to provide positive feedback

Several participants explained their motivation for commenting on a friend’s post as an act of altruism to boost others’ self-esteem, stimulating peer connectedness through provision of emotional support and mutual respect (F4, F5, F10). However, others conveyed a weight of expectation to do so to avoid negative consequences to the friendship. Failure to like or comment on pictures posted by friends was usually met with confusion (‘because it’s the normal thing to do’ (F11)), a need for justification, or conflict (M4).

While many participants accepted this etiquette as part of everyday peer relationships, others described it as time-consuming and ‘overwhelming’ (F15), with some feeling ‘forced’ (M4) to like or comment on a friend’s post. This emotive language seemed to convey a sense of excessive peer connectedness or over-stimulation emerging from unrealistic but increasingly normalised expectations of friendship.

Participant F12 described the process of commenting and liking on others’ posts as ‘trading’ to boost perceived popularity for enhanced peer status. This understanding that provision of positive feedback is expected rather than based on genuine positive evaluation of content may undermine the validating effect of receiving positive feedback oneself, leading some young people to view likes or comments received on their own content as a superficial form of popularity and undermining benefits to self-esteem.

In addition to extending the positive stimulation effect of online communication into negative feelings of oppressiveness, the concept of displacement is exemplified here through young people’s defining of friendship in the normative obligation to exchange likes and positive comments. These more potentially hollow popularity-based aspects of friendships are indicative of ‘weak’ ties – a superficial type of peer support compared to the deeper benefits of strong affective ties defined by close emotional, tangible support, mutual respect and trust [11]. However, peer popularity is a key aspect of identity development and sense of self in adolescence, and receipt of feedback to social media content may therefore still be an important contributor to well-being and peer connectedness for this age group [27].

#### (Mis)trust

The theme of (mis)trust encapsulated both positive and negative aspects of the role of SMU in social relationships. The dominant narrative presented social media as a vehicle through which participants’ parents could demonstrate their trust that they would behave safely and responsibly. This was generally reciprocated by participants, several whom trusted their parents or other family members to follow their social media accounts as a form of protection. One exception to this provided an example of a more complicated relationship with parents and felt a lack of trust to be left in charge of their own SMU, with implications for responding to adverse online experiences.

Where close friends were felt to be trustworthy, social media provided opportunities for self-disclosure, fostering intimacy in the relationship, and improving peer connectedness in a virtuous cycle. However, the fear of data misappropriation, such as screenshotting within broader peer networks, appeared to have led to widespread underlying feelings of mistrust, undermining peer connectedness. Rather than a linear effect of displacement or stimulation, displacement or undermining of social connectedness seemed to present in a poor-get-poorer effect, whereas good quality relationships were further stimulated by SMU in a rich-get-richer effect.

#### Opportunities for adults to demonstrate trust

With adolescents in control of their own online profiles and content, social media was felt by some to provide opportunities for adults in their lives to demonstrate they trust young people to be responsible online, nurturing their independence. Within the sample, there were positive examples of trusting parental relationships, in which parents had provided guidance and established boundaries, then let young people use social media without excessive interference (M1, F8, F10).

Several participants spoke of their parents or other family members following their social media accounts to keep an eye on them. This was generally framed positively as overseeing participants’ SMU for their protection, either in terms of giving advice about data privacy or inappropriate posts (F17), or in more practical terms, whereby geographical tags can help parents locate young people if they are unable to contact them (M4). Participant M4 also went on to discuss the barriers introduced by social media to prevent lying to his parents about his whereabouts. This was also framed positively as preventing potential damage to the relationship. The dominant narratives of mutual trust developing and being played out through parents’ navigation of young people’s SMU demonstrated the potential for stimulation of family connectedness.

However, one participant stood out in their portrayal of parents with strict attitudes to SMU, whereby access to certain apps or activities had been banned, describing a paternal relationship defined by restrictions and lies.

*My mum gave [snapchat]to me when I was 10, and then my dad said I wasn’t allowed to have it. I kind of deleted it for a while, and then I discovered I could just hide it, so I had it on my phone. Then whenever he asked to use my phone, I’d delete it, and then download it again and put it back in when I got my phone back… (F1)*

This participant also spoke of her parents looking over her shoulder as she used social media or taking her phone out of her hands to check what she was doing.

Those participants with parents who had demonstrated their trust reported feeling able to discuss and ask for advice on difficult issues encountered on social media, whereas those with less trusting parents felt reluctant to approach their parents for fear of repercussions. This is evident in contrasting comments from F14, who was comfortable approaching their mother for help with online peer relationships, and F1, who felt that her parents’ dogmatic approach to social media prevented her reporting online sexual harassment in case she was no longer allowed to use certain apps.

*I mean she [mum] knows that you’re going to get follow requests from people you don’t necessarily know and she said, “You can accept them but just make sure you know what you’re getting into.” She’s like, “If anything gets too bad tell me because we’re not going to tell you off or anything. We want to understand and even if you’re in the wrong we’ll try to help you”. (F14)*

*But I wouldn’t tell my parents [about strangers’ sexual harassment online] because they wouldn’t let me have it any more, and I’m not really meant to have it anyway. (F1)*

Participants who had established a sense of mutual trust with parents also noted an appreciation for constructive guidance and boundaries to SMU. This appeared particularly pertinent to night-time SMU and its potential to disrupt participants’ sleep patterns, where rules set by parents about SMU in bed were quickly found to be beneficial by participants F4 and F5. In this sense, an authoritative approach to setting sensible SMU boundaries – seemingly reflective of good family connectedness – seemed to be acceptable to young people. Family connectedness therefore has the potential to mitigate well-documented negative effects of night-time SMU on sleep [14].

#### Self-disclosure and fear of screenshotting (‘I don’t trust you’)

We found some evidence of online enhanced self-disclosure in our sample – in line with the stimulation hypothesis – whereby features of online communication facilitate sharing of intimate information, leading to better quality relationships [28]. Those participants who demonstrated online enhanced self-disclosure appeared to do so specifically because of perceived poor social skills. Participant F6 described herself as particularly lacking in social confidence and noted a preference for sharing sensitive disclosures via social media rather than at school where ‘everyone is always there’ (F6). In this case, the perceived privacy of direct messaging via social media with trusted close friends was felt to stimulate online self-disclosure and deepen the participant’s friendships. M1 also noted difficulties approaching friends face-to-face with a problem, but an ability to be ‘direct’ in doing so online. One participant gave a specific example of preferring online rather than face-to-face interaction in the case of a close bereavement, where giving condolences online would avoid an uncomfortable display of emotion (M4).

However, a more common perspective amongst our sample was a preference for face-to-face sharing when it came to sensitive or personal information. Reasons included the increased effort involved in typing long messages online (F5), ease of conversation and avoiding misunderstandings when able to gauge behavioural or vocal cues (F4, F7, F10, F11, F12, F14, F15, F18, F19), knowing who else is present and increased privacy offline (F2, M2, M3, M5, F8, F16, F17), and face-to-face as a less superficial and therefore more appropriate context for discussing serious problems (F9).

**Fig. 1:**
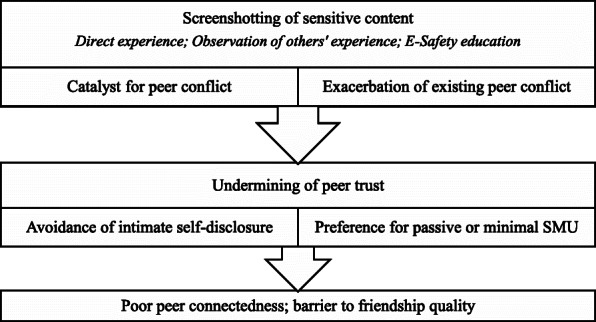
Implications of social media screenshotting for trust and poor peer connectedness

For many participants the risk of screenshots being taken and shared presented a substantial barrier to online self-disclosure (Fig. 1), with some saying they would not trust even close friends with sensitive information sent over social media. Others reserved any content sharing only for trusted close friends, and only using certain apps such as Snapchat where users are notified if someone has taken a screenshot. Fears included screenshots being used as ‘evidence’ (F13) or ‘proof’ (F15) within an argument, or to spread ‘rumours’ (F6), but also images being manipulated and used to ‘make fun’ of the subject (M3). Other participants blamed screenshotting for exacerbating peer conflict and for the potential ‘break[down]’ (M5) of friendships. For two participants (M2, M3), the risk of screenshotting and potential misappropriation of content put them off using any social media at all. In restricting online self-disclosure, this fear and mistrust of social media audiences represent a limit to online peer connectedness, aligned to the displacement of good quality face-to-face social interactions with less intimate ones online.

Concerns about deception and privacy issues appeared to be at the forefront of most participants’ minds as a result of their own or peers’ experiences, or anxieties raised by parents. These worries ranged from trusting (or not) their friends to sensitively handle content shared privately, feeling ‘suspicious’ (F8) when contacted by strangers as to their identity and intentions, to a general undercurrent of mistrust of social media audiences not to ‘hack’ (M4) their accounts, ‘steal’ (F18, F19) their data or identity or engage in other ‘scary’ (F2) behaviour.

*Probably if I had to think of something off the top of mind, I would probably say the most important thing on social media is, don’t talk to someone you don’t know, because you don’t know what they’re capable of. (M4)*

The young people in our sample were thus acutely aware of the risks of identity theft and of engaging online with potentially dangerous strangers. Combined with a general discomfort with online self-disclosure or fear of screenshotting among peers, this mistrust can simultaneously be perceived as a challenge to quality in peer relationships and interpreted as a constructive strategy for mitigating risk.

### Personal and group identity

Social identity development and expression can be facilitated through SMU. Using social media to share experiences – messaging, viewing online content together with friends and family, and co-producing content such as TikTok videos – appeared to foster feelings of connectedness through stimulation of a sense of belonging. Participants described careful curation of their online profiles to construct and express their identity. Online social networking and microblogging enabled those with specific interests (such as art or music) or experiences (including mental health conditions) to find like-minded others and join communities without geographical constraints, thus enhancing peer connectedness.

In terms of family connectedness, frustrations with adults’ lack of understanding of young people’s SMU and overemphasis on online harms led to a perceived disconnect between generations. Rather than displacement weakening family connectedness here, an adult discourse of SMU displacing activities they perceived to be healthier had negative implications for highlighting differences between generational groups and reducing feelings of mutual respect and understanding.

#### Sense of belonging

SMU stimulated feelings of social connectedness via enhancing feelings of belonging and group membership. For some participants, this was achieved simply through inclusion in a group chat (F3, M4).

In other cases, appearing on Instagram stories, ‘slip stories’, or private stories of their friends – whether as actors within the content or as privileged audiences of this restricted content – helped to cement participants’ position as a trusted member of the peer group (M4) and was generally perceived to symbolise a close friendship (F1, F2, M4, F9, F11). In such cases, privacy settings became markers of group membership.

In line with the stimulation hypothesis, SMU enabled and made salient shared experiences with existing friends and family, an important part of social group membership. Several participants highlighted the shared enjoyment of passively consuming social media content in the presence of others. This included watching YouTube videos together with family members (M3), sharing funny memes with parents (M1, F8), and using content related to special shared interests (such as football) to enrich interactions with siblings and improve the closeness of the relationship (F6). In addition, active co-production of visual content with others was presented as an important part of friendship for some (F9) and a way to ‘make memories’ with friends (F5). This co-production could improve peer connectedness through collaboratively working to achieve a common creative goal and sharing in a sense of accomplishment.

For some participants (F18, F19, F2), social media represented an opportunity to express their opinions and share creative projects with like-minded others outside their immediate friendship groups, with whom they would otherwise be unlikely to interact because of differences in age or location. Using social media in this way gave them access to communities in which they could receive support in shaping their artistic identities as well as becoming active and supportive community members. Identity development and expression was thus supported by SMU, simultaneously stimulating connectedness to a wider peer network.

#### Generational disconnect

Many participants expressed a sense of frustration with what they perceived to be an adult obsession with screen-time and the negative effects of SMU, which seemed to impact negatively on family identity and connectedness. Growing up in a vastly different environment to their elders – largely but not exclusively related to the advent of social media – was felt to have led to a disconnect between generations, whereby adults were perceived as unable ‘to relate’ (F10, F14, F15). As such, there was a sense that adults fail to fully appreciate the significance of the online world for this generation, imposing arbitrary screen-time limitations rather than taking time to understand the positive and negative aspects of SMU.

Older generations were felt to overestimate the negative impacts of SMU (‘adults think it’s bad but it’s not that bad…’ (F13)) with too much importance placed on social media as a cause of bullying or harm, when the relationship as experienced by young people, is more complex.

*I think the biggest problem with social media is adults say, “It’s evil, you shouldn’t do it,” but the thing is- and they’re like, “It creates argument, you bully each other.” It doesn’t. The thing is the arguments are going to happen anyway, it just doesn’t help you resolving it really. People are like, “Oh it creates arguments. It turns people into bullies. You’re vulnerable on there.” It isn’t really. That’s the thing. (F13)*

Several participants relayed experiences whereby their parents or other family members had been critical of their SMU, with a general negative ‘stigma’ attached to social media (F10). While this was sometimes perceived as a justifiable concern around online harms (F4, M4), those who were told to simply ‘get off your phone’ (F12) felt misunderstood and some found this irritating or upsetting (F2, F10, F11, F12, F14, F15). Participant F14 described her mother’s dismissive attitude towards social media. Her mother suggested that her SMU displaced time better spent on healthier activities such as exercise and face-to-face socialising, but was felt to underestimate the social importance of SMU and the diverging priorities between generations. With social media often used strategically at times when such activities are logistically more difficult (as discussed under ‘displacement of face-to-face socialising’), this perceived inappropriate emphasis on displacement and screen-time restrictions appeared to underlie the sense of disconnect between young people and older generations. These age-related group differences – accentuated by divergent attitudes to SMU – have the potential to increase inter-generational discord, harming family connectedness through diminished feelings of mutual understanding and respect.

## Discussion

This qualitative study contributes to a growing literature on the psychosocial impacts of SMU in adolescence. We explored in depth the role of SMU in the broader social environment from the perspectives of adolescents themselves, examining both peer and family connectedness. Four themes were identified: i) ‘Displacement of face-to-face socialising’ (ii) ‘Social obligations’ (iii) ‘(Mis)Trust’ and (iv) ‘Personal and group identity’.

### Findings in relation to displacement and stimulation hypotheses

We found some limited evidence in favour of the displacement hypothesis [11], whereby time spent using social media was felt by some participants to displace time spent socialising with family or friends face-to-face. However, it was often the case that online peer interactions took place mainly when in person socialising was not possible, providing opportunities to socialise and maintain peer relationships online in the absence of offline opportunities. Those experiencing increased SMU in place of family socialising tended to relay lower levels of family connectedness that preceded the SMU, with SMU used strategically to overcome feelings of loneliness in the home. This supports a ‘poor-get-poorer’ or ‘social deterioration’ effect, whereby those who feel less connected to their family are likely to rely more on SMU for social interactions or to alleviate boredom at home, further compounding a lack of connectedness within the household. Considering peer and family connectedness together, this is also illustrative of a ‘poor-get-richer’ or ‘social compensation’ effect, whereby poor family connectedness leads to increased online socialising with friends and subsequent improved peer connectedness. SMU may therefore serve as a protective tool in some circumstances to mitigate psychological risks associated with poor family connectedness or reduced face-to-face socialising. It is worth noting that these interviews took place before the COVID-19 pandemic led to school closures and lockdown, and SMU is likely to have served a particularly important function in this regard over the course of the pandemic.

One of few studies examining SMU and family connectedness, a cross-sectional survey of Canadian adolescents [29] found that heavy SMU (3 or more hours per day) was associated with greater odds of negative reported relationships between mothers and daughters, fathers and daughters and fathers and sons, but not mothers and sons. The authors explain their results as indicative of SMU displacing time spent engaging face-to-face with parents, with negative consequences for family relationships, However, our findings indicate that adolescents may also be motivated to turn to social media as a result of existing poor family connectedness.

Our study provides more evidence for the stimulation hypothesis, whereby SMU enhances the user’s existing social resources through increased contact and maintenance of relationships. Perceived benefits of SMU that emerged in this sample included the expansion of social networks, the ability to keep in touch with friends and family (including those for whom geographical constraints prevent offline socialising), enhanced self-disclosure for socially awkward young people or among very close friends, and supporting identity development and feelings of belonging. Consistent with a ‘poor-get-richer’ effect, those with reduced social resources offline – not only due to social awkwardness or anxiety but also loneliness or geographical barriers to offline interaction – find online support particularly beneficial [17, 27].

**Fig. 2:**
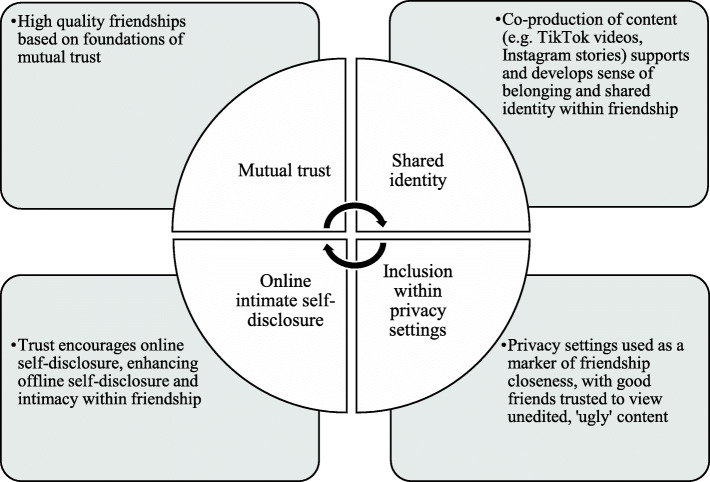
Social enhancement (rich-get-richer) effect of social media in connectedness within close friendships

Where close friends were felt to be trustworthy, social media provided opportunities for self-disclosure, fostering intimacy in the relationship and improving peer connectedness in a ‘rich-get-richer’ or ‘social enhancement’ effect (Fig. 2). This is in line with previous research finding that adolescents’ time spent on instant messaging services enhances time spent face-to-face with friends, and subsequent quality of friendships [20]. SMU also provides opportunities for young people to construct, express, and develop identity in relation to their social world [30]. Young people in our sample reported using social media to share experiences, such as passively watching entertaining content together with friends and family members, as well as actively co-producing content, with privacy settings used to demarcate friendship group boundaries to different degrees of closeness (Fig. 2). This may foster feelings of connectedness and belonging, in line with the stimulation hypothesis.

In addition to the positive aspects of SMU, young people reported feeling pressures of expectation around providing feedback on friends’ online posts and being constantly available for communication. For these young people, social media had created a normative environment of ‘over-stimulation’, which fostered feelings of stress. It may be that SMU for direct peer communication may stimulate connectedness and subsequent well-being to a point, whereas excessive communication and the associated expectations to respond might undermine these benefits. This aligns to the 'digital Goldilocks hypothesis' [24] and other evidence of a curvilinear relationship between SMU and psychosocial adjustment [23], whereby moderate SMU is beneficial to well-being (compared to no use at all) but excessive use is associated with negative outcomes. In addition, the fear of data misappropriation such as screenshotting within broader peer networks appeared to have led to widespread underlying feelings of mistrust, thus undermining peer connectedness, and lending weight to the suggestion that broader SMU may discourage development of ‘strong ties’. Screenshotting is a currently understudied aspect of SMU. Our findings suggest the role of screenshotting within relationships between SMU and psychosocial outcomes – including social connectedness – warrants further attention.

### Parental understanding of social media use in young people

**Fig. 3:**
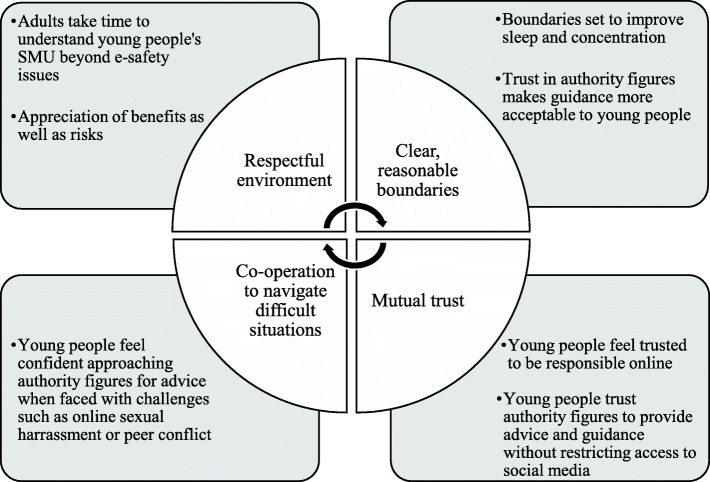
Social enhancement (rich-get-richer) effect of social media in family connectedness

Where a family environment of mutual respect had been established and consideration had been given to understanding the indispensable role of social media in young people’s lives, with positive aspects acknowledged in addition to traditional e-safety concerns, young people were more accepting of advice and clear boundaries regarding healthy SMU. Young people felt they were trusted to behave responsibly online and in turn trusted authority figures to provide guidance regarding challenges encountered without fear of access to social media being removed or restricted. Risks to peer connectedness encountered online, such as cyber-ostracism or screenshotting, may thus be mitigated by strong family connectedness. With this supportive environment, young people are able to navigate online difficulties but also feel encouraged to share positive social media content with family members, promoting shared interests and family identity. A ‘rich-get-richer’ [19] effect appears to develop, with social media promoting further trust and family connectedness (Fig. 3).

**Fig. 4:**
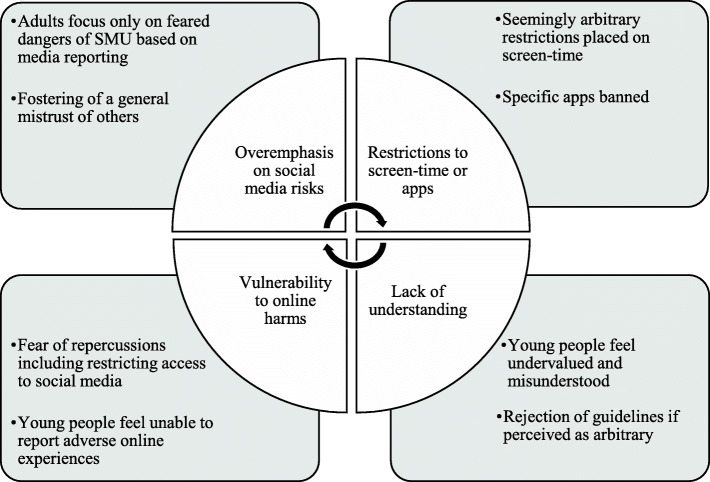
Social deterioration (poor-get-poorer) effect of social media in family connectedness

Conversely, an existing lack of trust in relationships between young people and their parents may underpin a rejection of screen-time restrictions and a reluctance to report exposure to online harms, adding further to a sense of social distance and further undermining connectedness, consistent with a ‘poor-get-poorer’ effect (Fig. 4). Future research might explore whether these findings can be generalised to the wider population of young people, and test the relationship between parental attitudes to social media and young people’s resilience or vulnerability to online harm.

### Limitations

This study has some important limitations. Our sample size was somewhat smaller than planned due to the emergence of COVID-19, with boys in particular under-represented. However, a broad range of views and experiences were captured in the sample, which were sufficient to enable rich themes to be generated [31]. While our sample was diverse in their experiences of social media, they were not selected on the basis of how much they used SMU or for what reasons, therefore it is possible that some additional patterns of SMU may exist in this age group that were not captured. All participants were aged 13–14-years and attended inner-city secondary schools in one area of the country. Different offline experiences and circumstances are likely to be accompanied by different online experiences, and caution should therefore be exercised in generalising findings from this study to other populations.

### Implications

The separation of the social environment offline and on social media is not clear cut. Focusing on developing trusting, attentive relationships with peers and parents offline is likely to optimise the potential for social media to further benefit social connectedness. Feeling understood and respected by adults should encourage young people to accept and appreciate healthy boundaries established regarding their digital activities. A balance must be sought between teaching young people about the risks to well-being that engaging with social media may lead to, without being alarmist and creating a culture in which confidence in others is discouraged. Healthy peer relationships in which there is trust, respect, and space to ignore digital notifications and messages are likely to benefit most from SMU that enables enhanced self-disclosure and increased closeness without feeling oppressive. Young people should be supported to re-prioritise trustworthiness over availability in defining meaningful and fulfilling friendships. If, as our evidence suggests, the online social environment is an extension of relationships in the real world, fostering healthy connectedness with others offline is likely to maximise the social benefits and minimise the potential harms of social media for young people.

## Conclusions

Rather than a clear, unidirectional relationship in which SMU harms – through a process of displacement – or enhances – through stimulation – overall social connectedness in adolescence, we suggest a complex set of reciprocal and circular relationships in which social media can play both a beneficial role in reinforcing existing positive connections to peers and family, and a deleterious role in exacerbating an already poor social environment through the propagation of mistrust. The relationship between SMU and social connectedness cannot be viewed as independent of either content or context. In addition to quality of existing offline social resources, the different activities and ways in which adolescents use social media will partially determine the direction and valence of effects. Passive SMU, devoid of social interaction, is unlikely to confer the same social benefits as SMU for direct communication with friends. However, parents and other adults supporting young people should also take account of individual differences in how social media may benefit or undermine connectedness, supporting individuals to find ways to interact with social media that best supports their well-being.

## Supplementary Information

**Additional file 1.** Social media use and social connectedness: interview topic guide.

## Data Availability

The qualitative datasets generated and analysed during the current study are not publicly available due to the data containing information that could compromise research participant privacy but are available from the corresponding author on reasonable request.

## Abbreviation

SMU: Social media use
