# Supplementary material for: Social media use and social connectedness among adolescents in the United Kingdom: a qualitative exploration of displacement and stimulation
Source: BMC Public Health. 2021 Sep 24;21:1736. doi: 10.1186/s12889-021-11802-9 (PMC8464110; doi:10.1186/s12889-021-11802-9)
Supplement: Supplementary file 1 — Additional file 1. Social media use and social connectedness: interview topic guide. [file 12889_2021_11802_MOESM1_ESM.docx]

**Social media use and social connectedness among adolescents in the United Kingdom: A Qualitative Exploration of Displacement and Stimulation; topic guide for year 9 students**

Introduction/ground rules

- Remind participants of confidentiality
- Remind participants they are free to take a break or stop at any time
- Please listen to and respect each other’s views if in a paired interview
- We are interested in all views, even if they are different from your friend’s – there are no right or wrong answers

Experience of Social Media

- What do you understand by the term ‘social media’? What are the first things that come to mind?
- How old were you when you first started using social media and which apps did you use first? Which do you use now?
  - How many different accounts do you have (spam accounts?)? Do you use them differently?
- What kind of things do you use social media for? Prompt: posting, selfies, comments, scrolling, finding info...
- Do you use it more or less at certain times or in certain situations? When do you use SM? Prompt: how about yesterday, talk through use that day
- Do you use it mainly at the same time as doing other things, or do you spend time just using social media and not doing anything else?
- How do you feel while you’re using it? How do you feel afterwards? Does it make you feel good or bad?
- **Is there anything you would like to change about your own social media use? Why/ why not?**
- Think about when you’re at home. Does SM affect the time you spend with family in any way? Are there any rules about use? Are they good or bad?
- Think about school. What are the rules? Do you think they’re good or bad? Homework? Concentration?
- Do you think girls and boys use SM differently?
- Do you think LGBTQ teenagers use SM differently?

Social media and mental health

- What sorts of things do you think of when we talk about mental health?
- (IF NOT ALREADY MENTIONED): Do you think how young people use social media can affect their mental health or how they feel about themselves? *Y/N? Why? In what ways? This can include positive and negative effects. Do you think it’s different for girls and boys, LGBTQ teenagers, different age groups, teenagers from higher/lower income families?*

**Closing question for all**

- Is there anything else you want to say about social media and mental health? Briefly summarise.
